# Mental Health Staff Perspectives on Spiritual Care Competencies in Norway: A Pilot Study

**DOI:** 10.3389/fpsyg.2021.794165

**Published:** 2022-02-18

**Authors:** Pamela Cone, Tove Giske

**Affiliations:** ^1^School of Nursing, Azusa Pacific University, Greater Los Angeles, CA, United States; ^2^Faculty of Health Studies, VID Specialized University, Bergen, Norway

**Keywords:** spirituality, spiritual care, competencies, mental health, nursing, mixed-method

## Abstract

Spirituality and spiritual care have long been kept separate from patient care in mental health, primarily because it has been associated with psycho-pathology. Nursing has provided limited spiritual care competency training for staff in mental health due to fears that psychoses may be activated or exacerbated if religion and spirituality are addressed. However, spirituality is broader than simply religion, including more existential issues such as providing non-judgmental presence, attentive listening, respect, and kindness (International Council of Nursing [ICN], 2012). Unfortunately, healthcare personnel working in mental health institutions are not well prepared to address spiritual concerns or resources of their patients (Cone and Giske, 2018). Therefore, a mixed-method pilot study was conducted using a self-assessment survey tool to examine spiritual care competencies of mental health staff in Norway and to understand the perspectives of mental health staff in the Scandinavian context (Stockman, 2018). Five questions and comments related to survey items provided rich qualitative data. While only a small pilot with 24 participants, this study revealed a need for spiritual care educational materials targeted specifically for those who work in mental health, materials that address the approach of improving attitudes, enhancing skills, and increasing knowledge related to spirituality and spiritual care of patients.

## Introduction

According to Nolan et al. (2011), the spiritual domain is multidimensional, and it includes existential concerns, value-based issues, and transcendent considerations, including religion, faith, and view of life. Unfortunately, there is limited knowledge about the perspectives of those who work in mental health facilities. Spirituality is a large umbrella term accommodating existential phenomena such as love and connectedness (Rykkje et al., 2015), meaning and purpose (Balgopal and Montplaisir, 2011), and hope (Harley and Hunn, 2015), as well as transcendence (Walsh, 2010; Pargament, 2013; Weathers et al., 2016). However, many still associate spirituality only with religion (Saleem et al., 2014), particularly in Norway where this pilot study took place. The association between spirituality and religion is especially so in Scandinavian countries where there is a long history with Christianity as the dominant religion and the term has elements of the word for religion within it. Furthermore, even though the spiritual domain includes far more than religion (Nolan et al., 2011; Skevington et al., 2013; Weathers et al., 2016), many mental health clinicians avoid spiritual and existential concerns because of the fear of activating a religion-related psychosis (Koslander et al., 2009; Ouwehand et al., 2014; Borge and Maeland, 2017; Neathery et al., 2020).

In their phenomenological study of spirituality in mental health, Tokpah and Middleton (2013) note that “spirituality is central to but forgotten in psychiatric nursing practice” (p. 81), which supports the earlier work of Swinton (2001). These findings are echoed by Norwegian researchers who point out that even though a person-centered, and whole person approach is being promoted in mental health care, spirituality is not yet a legitimated theme (Borge and Maeland, 2017). A large study done among psychiatric mental health nurses in the United States reported that the “most frequent barriers to providing spiritual care were lack of education and fear of exacerbating psychiatric symptoms” (Neathery et al., 2020 p. 370). There is a demonstrable need for more professional education on spirituality and spiritual care in mental health care because health care professionals working in this field lack competences in addressing spiritual concerns of patients (Helminiak, 2001; Saleem et al., 2014; Medås et al., 2017; Patterson et al., 2018; Neathery et al., 2020).

There are many sources of research showing an association between faith and positive health benefits such as coping with illness and faster recovery (Koenig et al., 2012). From a Scandinavian point of view, there have been critical voices to the mainly American research around faith and health due to the differences between how faith is practices and lived out in Scandinavia (Hvidt et al., 2020). Even though Scandinavia has 1,000 years of Christian history and most have had Lutheran state churches until recently, Scandinavian countries are seen as some of the most secular societies in the world (Hvidt et al., 2020). Although the majority of the population in Norway belongs to a Christian denomination (68% belongs to the Norwegian Church, and 7% to other Christian denominations [Statistics Norway, 2021a,b], church attendance is low, and it may seem like people live passive religious lives; thus, it is almost taboo to talk about religion (Giske and Cone, 2015; Kuven and Giske, 2019; Hvidt et al., 2020), which also means the many Scandinavians do not have a language to talk about their spiritual concerns.

Holmberg et al. (2021) point to the need to develop spiritual literacy amongst professionals in Norway, which is also discussed by others (Giske and Cone, 2015). However, when facing crisis and illness, many Scandinavians start thinking about God and reflect about their situation (Hvidt et al., 2017). Hvidt et al. developed two concepts that might be helpful in discerning what meaning religious faith might have in people’s lives and how helpful their faith is for them in times of illness. “Restful religiosity” is used about people who rest intrinsically in their faith, where faith is internalized through identification and the person deeply hold the values of the religion. “Crisis religiosity” is used about people where challenging life events such as crises and illness increase their reliance on religion (Hvidt et al., 2017, 2020). Hvidt et al. (2017) ask the question if crisis religiosity has become the predominant form of religious expression in the Scandinavian population.

Koslander et al. (2020), also reporting from Scandinavia, found that patients who had been inpatient in psychiatric care described their experiences of spiritually as going beyond religion, even though religious experiences were part of it. Spirituality could provide resources such as hope, connectedness, meaning, and coherence in life, but also give rise to doubt, anxiety and feelings of loneliness, and hopelessness. Koslander et al. (2020) therefor advise mental health nurses to approach spirituality as a dialectic matter and being open to their patients, as spirituality often is in a dialectic relationship to each other as both a resource and a challenge. In one study exploring the relationship between religious and spiritual needs that are met versus unmet needs, van Nieuw Amerongen-Meeuse et al. (2020) found that it is very important for nurses and other mental health practitioners to give personalized attention to religion and spirituality in their conversations with patients.

Recently there has been more research related to the lack of preparation nurses report for addressing the spiritual domain with their patients (Ross et al., 2018) and the strategies being used to address this issue (Rykkje et al., 2021). A large study by van Leeuwen and Schep-Akkerman (2015) exploring nurse perceptions of spiritual care in Netherlands revealed that there are significant differences between health care settings (hospital care, mental health, home care) in almost all areas measured. They recommend that “nursing practice, nursing education, and nursing management should consider an emphasis on spiritual competence development related to working settings of nurses” (van Leeuwen and Schep-Akkerman, 2015, p. 1354). Moreover, even though mental health services currently focus on a recovery-oriented, person-centered approach, the spiritual domain is often neglected (Davies et al., 2019), and overall, there is limited research and specific education globally addressing spirituality and spiritual care in mental health facilities (Tokpah and Middleton, 2013; Saleem et al., 2014; Bitter et al., 2020).

This pilot study, planned and implemented by the authors as co-Primary Investigators (co-PIs), had two aims. First, we aimed to evaluate use of the Tool among mental health staff, and secondly to describe the views on spirituality and spiritual care of healthcare personnel working in a Norwegian mental health institution and to identify their knowledge, skills, and attitudes related to spirituality and spiritual care of patients in their workplace.

## Materials and Methods

### Participants

This mixed-method pilot study was conducted at one mental health hospital in Western Norway by two researchers, one American and one Norwegian (the authors). Ethical approval for the study was discussed with Norwegian Data Protection Services. Since no personally identifiable data were to be gathered, informed consent was implied by a willingness to participate and staff returning the filled in survey to hospital leaders. A convenience sample of health care staff from five different wards were invited to take part in the survey; it must be noted that due to the pandemic, researchers were not able to do recruitment personally. Twenty-four healthcare personnel from different professional backgrounds (nurse, social educator, nurse assistants, aides) returned the survey. Although several ward leaders were approached to help with recruitment, half of the answers came from one ward where interest in the topic was high. Demographic data are found in Table 1.

**TABLE 1:** Demographic data.

Category	Variables and values				
Profession	BSN = 12	BSSocEd = 5	Other = 7		
Further education	Yes = 7	No = 15	Missing = 2		
Gender	Male = 6	Female = 17	Missing = 1		
Wards of work	S1 = 12	4 Others = 12			
Age in decades	20 s = 11	30 s = 8	40 s = 4	50 + = 1	
Years working mental health	Less than 2 = 4	2–5 = 9	6–11 = 3	12 + = 7	Missing = 1

### The Survey

The survey focused on spirituality in nursing care. The survey instrument is a recently developed and tested EPICC Spiritual Care Competency Self-Assessment Tool, hereafter called the ‘‘Tool,’’ with parametric testing that showed its validity and reliability as an instrument. Developed from the EPICC^1^ Spiritual Care Educational Competence (SCEC) Standard for bachelor students (McSherry et al., 2020; van Leeuwen et al., 2020), the Tool provides an opportunity to personally assess one’s competencies, based on the elements of the EPICC SCEC Standard, and to evaluate one’s growth when used again over time. The Tool includes 28 questions where participants can score their knowledge, skills and attitudes on a 5-point Likert scale addressing four areas of spiritual care competencies; (1) Intrapersonal spirituality, (2) Interpersonal spirituality, (3) spiritual care Assessment and Planning, (4) spiritual care Intervention and Evaluation. During its initial parametric testing after being developed, the Tool scored a very high Cronbach *alpha* (0.910), so it was determined to be reliable, and its validity was sound (Giske et al., 2022).

There are six demographic elements in the survey: profession, age, gender, further education, years working in mental health, and the ward where they primarily worked. After filling in the EPICC SCEC, participants were invited to write a short reflection regarding their own spiritual care competences. There are also five ending questions, two of which can be self-rated on a 1 to 5 Likert scale: (1) how important it is to be able to conduct a spiritual assessment, and (2) how well prepared they feel to conduct a spiritual assessment. Three other questions address the following: (3) have they had had any education in conducting a spiritual assessment, (4) how do they collect patient information to carry out a spiritual assessment, and (5) have they had experiences in supervising nursing students in following up patients spiritually. Four of these questions have a quantitative format as well as room for comments, so those numerical answers were added to the survey dataset for quantitative analysis. Lastly, participants were invited to share further comments if they wished. All written comments were analyzed qualitatively.

### Data Collection and Analysis

The leaders of the hospital agreed to take part in the pilot, which is the first part of a bigger study where a Ph.D. student is conducting interviews regarding spiritual care with patients and later with staff at two of the wards in the hospital. The Norwegian co-PI delivered the survey to one of the nurse leaders who distributed them to five of the hospital wards. The completed questionnaires were placed in an envelope to be picked up by the co-PI (second author) after 5 weeks, in February 2021. We had hoped to have a higher number of surveys returned, but Covid-19 restrictions prevented us from personally recruiting or discussing the pilot with staff ourselves, so we had to rely on the identified gatekeepers. Reasons for the low response rate were later explained by the nurse leaders as due to two other studies going on at the same time, but the low numbers may have more complex reasons than that. Since this survey was a pilot study in this mental hospital where no spiritual care research had yet been done, we felt it warranted a voice in the research literature; hopefully, future research will be done on spiritual care in this and other mental health institutions in Scandinavia.

Data from the surveys were entered into a spreadsheet that was then imported into quantitative software, and the open comments were translated into English by the Norwegian co-PI for discussion and analysis by the two co-PIs. Of the questions at the end of the survey, numbers 1, 2, 3, and 5 were easily quantified and added to the SPSS dataset. The American co-PI analyzed the quantitative data using SPSS version 28, and a number of tests were run: *t*-test, ANOVA, chi-square, correlation, and factor analysis. The open comments from the survey as well as questions 3, 4, and 5 were analyzed using reflexive thematic analysis (Byrne, 2021). The co-PIs/authors used open coding followed by selective coding to analyze the comments separately, and we later discussed codes and themes together before agreeing on the final themes and sub-themes.

## Results

### Quantitative Results

The quantitative results of the survey include a look at the reliability of the Tool in light of the resent psychometric testing done by those who developed the instrument. The American co-PI consulted with a statistician to make sure all tests were accurately done and evaluated. Additionally, the participant demographics, the four spiritual care competencies, and the four quantitatively focused questions were analyzed.

#### Demographics

The 24 participants answered the questionnaire with few areas of missing data. Every ward that was approached about the project had at least one or two participants, though one ward with an engaged leader provided half of the respondents. Additionally, 17 of the 24 had bachelor degrees in nursing (BSN) or in social education (BSSocEd), so the group had a high level of education as a whole. Only a quarter were male, but nursing is generally a female dominated profession, so that is not unusual. Most did not have preparation or training for spiritual care, and had uncertainty about how important it is in the care of mental health patients. While we asked for their age, we entered data as decades, and most were young adults (20–39 years old).

#### Evaluation of the Tool

While this pilot study had a small sample size (*n* = 24), the co-PIs thought it would be good to examine the psychometric results and compare findings from this pilot among mental health nursing staff with results from the original parametric testing of the Tool. Using the SPSS-28 software, the statistician consultant computed the overall Cronbach *alpha* (0.838) for the Tool used in mental health, which for a pilot study is very good. While the evaluation of the Tool in mental health showed a high *alpha*, we thought it would be interesting to do an item analysis on each of the four competencies. This revealed that each of the four competencies had one question that scored quite low compared to the others within the subgroup. For Competency 1—Intrapersonal Spirituality, question 6 (of 7) was a complex question on Attitudes that addressed multiple concepts. When removed from that first competency, the *alpha* score of that competency went from 0.548 to 0.638. Competency 2—Interpersonal Spirituality had a complex Knowledge question (#2 of 5) that addressed both awareness of and impact of knowledge; removal of that item raised the Cronbach *alpha* score on that second competency from 0.556 to 0.661. Competency 3—Assessment and Planning of Spiritual Care again had an Attitudes question that was complex (#7 of 8), in that it combined several attitudes rather than simply addressing one. The score there moved from 0.667 to 0.699, a small but significant change. Finally, in Competency 4—Intervention and Evaluation of Spiritual Care, the second question in the Knowledge category (#2 of 8) was complex in that it addressed both patient needs and resources, which made it difficult to answer. The Cronbach *alpha* score moved from 0.677 to 0.724 on that last competency. These issues will be raised with the SEP Team that developed the Tool in case there is a real need for modification of the Tool, which is seen in its complete form in the article by Giske et al. (2022), for future use among students and nurses working in a variety of fields.

#### Quantitative Analysis of Survey Questions

The survey Tool was created with four competencies based on the idea of knowing yourself (1-intra-personal) and how you relate to others (2-inter-personal), and then about learning how to assess and identify a problem or need and to create a plan of care (3-assess/plan), as well as how to intervene with compassion and to evaluate, document, and modify a care plan (4-intervene/evaluate) with attitude, skills, knowledge addressed in each competence. It is interesting to note that in all four of the competencies, the skills questions scored well in factor analysis. Some questions on Attitudes (#6 in Competency 1 and #7 in Competency 3) were difficult to understand (Cone and Giske, 2018), so they had a lower score, while two Knowledge questions (2nd question in both Competencies 2 and 4) were complex and multi-faceted, which made them hard to answer.

What is quite interesting is that the most significant and strongest relationship among these variables is the one between personal, intra-spirituality and the healthcare provider’s ability to intervene and evaluate the spiritual care given (*r* = 0.819, *p* < 0.001). Two other significant relationships between the competency scales that relate to intra-spirituality are with inter-personal spirituality (*r* = 0.582, *p* = 0.003) and with assessment and planning of spiritual care (*r* = 0.472, *p* = 0.020). This means that the more you know yourself and your beliefs, values, and what is important to you, the more able you are to facilitate spiritual care and to check and see if it was useful and successful or not.

It is interesting that the inter-personal spirituality element of spiritual care has a significant positive correlation to all the other scales, including the one to intra-personal spirituality mentioned above. Inter-personal spirituality also relates to spiritual care assessment and planning (*r* = 0.487, *p* = 0.016) as well as intervention and evaluation (*r* = 0.596, *p* = 0.002), which may, in some part be connected to the training in therapeutic communication that nurses receive and to the teamwork that nurses are encouraged to engage in when providing patient care (Institute of Medicine [IOM], 2010). Assessment and planning for spiritual care have only a moderately strong correlation to intra- and inter-personal spirituality, but these relationships are still very significant (*p* < 0.05). Attitudes, knowledge, and skills in spiritual care assessment and planning naturally relate to one’s ability to intervene and evaluate in a strong and significant way, which also makes sense, but it is good to see this confirmed.

### Questions at the End of the Tool

#### How Important Is Spiritual Assessment in Mental Health?

Due to the small sample size, no statistical significance was found with *t*-tests, though age revealed a difference between younger and older staff that approached significance. There was a relationship, again only approaching statistical significance, between bachelor-educated staff and the importance placed on the spiritual domain in mental health. This is also true of the question about how important spiritual assessment is and years of experience in mental health work. The ANOVA to determine differences between the importance of spiritual assessment and the four competencies showed that mental health staff who believe that spiritual assessment is important have a greater ability to work with each other (2-Interpersonal) and to conduct spiritual assessment (3-Assess and Plan) than those who do not (see Table 2). These differences were highly significant (*p* = 0.022 and *p* = 0.029, respectively).

**TABLE 2:** Importance of spiritual assessment—ANOVA.

ANOVA						
Model		Unstandardized coefficients		Standardized coefficients	*t*	Sig.
		B	Std. Error	Beta		
	(Constant)	–2.049	1.685		–1.216	0.240
	Intra-spirituality	0.226	0.576	0.112	0.392	0.700
	Inter-spirituality	1.171	0.492	0.500	2.379	0.029
	SC-assess and plan	1.034	0.409	0.521	2.528	0.022
	SC-evaluate and intervene	–0.896	0.677	–0.405	–1.324	0.203

*Dependent Variable: Q1-How important is it for nurses and mental health workers to do spiritual assessment?*

#### How Well Prepared Are You for Spiritual Assessment?

Chi-square tests revealed a linear association between years of work in mental health and a sense of preparedness for spiritual assessment, but most of the demographic elements do not have a significant relationship found through cross-tabulation of data due to the small sample size. When considering preparedness for addressing spirituality, it is interesting to note that there is a linear relationship between both groups of staff with bachelor other education as opposed to those with less or no formal training, which means that the more formal education you receive, the better prepared you are for spiritual assessment. The relationship between bachelor prepared nurses as opposed to other types of training approached significance with the sense of preparedness for spiritual assessment. This may be more related to the registered nurse’s overall training in assessment of the whole person than to any spirituality education, since only three participants noted that they had some level of training related to spiritual care.

#### What Training Have You Received for Spiritual Assessment?

Most of the participants noted that they had not received training in spiritual assessment. With only 3 of 24 responding yes, the lack of specific spiritual care education that prepares staff in mental health in this domain is clear, but no quantitative tests were useful in exploring this further. Qualitative findings shed some light on this element.

#### What Experience Have You Had of Supervising Students in Spiritual Care?

This final question would show significance with a larger sample size, but clearly, those with bachelor education in nursing or in social sciences and education (*n* = 17 of 24) are the ones chosen to supervise students in clinical practice. A sample of 17 participants with a degree is not enough to demonstrate statistically significant differences or clear relationships. We can simply infer, from linear associations, that the more education one has, the more experience you may have with addressing the spiritual in patient-centered, whole person care and/or supervising students. This is an example of holding to high standards for clinical education and student supervision, which is clear in this sample of mental health staff.

### Qualitative Results

Participants were asked one question to help them reflect on the tool and three other questions where they could comment on other aspects of spirituality and spiritual care. Reflexive thematic analysis (RTA) (Byrne, 2021) to identify and select codes was a useful approach to these open comments by mental health nursing staff. We will present our RTA findings for each of these questions, some of which had only a simple theme while others had sub-themes as shown below.

#### Personal Competencies

Staff were open and honest about reflecting on their competence in spiritual care after scoring themselves with the Tool. Fifteen of the 24 respondents addressed this question. Within their personal competence, four sub-themes emerged: the need for improvement, the environment in mental health, attitudes of healthcare staff members, and personal attributes of healthcare team members.

##### Need for Improvement

Half of the respondents who answered this question noted that they need improvement in the spiritual domain. A need for raised awareness was a common theme. Increased knowledge was also a need among staff. One noted that they are not religious and have little preparation for addressing the spiritual, while another reported being a “person of faith” and would like to improve being able to appropriately address such concerns with their patients.

##### Mental Health Environment

Many of the respondents note that their workplace is not the best environment for addressing the spiritual domain. Some say this is because many patients “have delusions related to religion or are in a crisis situation” so they need to be careful in how they address patients’ beliefs and life views. Others report that there is little spiritual care focus at work, with one stating there is “little focus on the theme in the context of where I work.” question.

##### Healthcare Team Attitudes

When considering the attitudes, skills, and knowledge approach to evaluating the spiritual domain with healthcare staff, it was interesting to note that awareness, knowledge, and skills in spiritual care need improvement, however, most respondents reported appropriate attitudes when considering the spiritual care of their patients. One reported respecting their patients and patient “views of life, spirituality, and faith.” Another noted always trying to “accommodate and facilitate for spiritual needs.” One noted that it is easier to address when their personal values match those of their patient. A few mentioned that “existential questions” are good to discuss with their patients. Finally, several reported being open and wanting to talk about what is important to the patient and about what will help them improve.

##### Personal Attributes of Staff

This sub-theme may be a factor that underlies the generally open attitude of healthcare staff reported in this study. A few noted that their “inherent personality” and their own professional achievements help them address difficult topics. One mentioned “being a person of faith” and said they were concerned that “I might find it good to talk about existential questions/life view/faith and wish to be open about talking about such themes with those around me.” Another shared “I use my personal experience from life in meeting people in crisis.” Others reported that they are a little “distant” from the spiritual domain and do not think about it either at work or elsewhere.

#### Previous Preparation

The three participants who reported that they had had training in spiritual care assessment provided open comments. One said they had gone to a faith-based bachelor’s program where there was an emphasis on spirituality and spiritual care. Two respondents noted that they read articles about the topic, and one had spiritual training unrelated to their profession. They do not explain what or where. It is interesting to consider whether the strong correlations found between the competencies in spite of a lack of preparation may be more related to their general nursing preparation than to any specific education in spiritual care.

#### Patient Assessment of Spirituality

Of the 24 participants who filled out the Tool, two participants reported that they do not assess spiritual issues at all, but 13 of them addressed the question on how they assess the deep, spiritual concerns of their patients. This is an amazing response in light of the lack of specific training in this domain. These staff appear to be very attuned to care of the whole person. Two sub-themes emerged.

##### Ways of Gathering Information

Within this sub-theme, communication was the key. Staff said they need to ask the patient directly about any issues that are spiritual in nature. This includes taking a history, observing carefully, listening well, and “mirroring” patient conversation so there is dialog where issues can be clarified for accuracy. Open-ended questions were seen as very effective and focusing on the future or next steps was a good approach with patients. Also mentioned is the location (not in public places) and timing (when they are alone with the patient) of these encounters.

##### Sources of Information

Participants reported that they use many sources in their assessment. The patient is important, but they also talk with colleagues and with family members to help them understand a specific patient issue, and some discussed reading materials that they searched for in books, articles, or on the internet. Documentation was mentioned as being another source of information. Talking with the priests and chaplains are also ways of gathering information about the patient and specific patient concerns.

#### Training Others

Only five of the 24 participants reported that they have had experience in guiding nursing students in the spiritual area, and they all commented on how they supervise students. Four of the respondents commenting were nurses, and one was a social educator. Two sub-themes emerged: Talking about the content and choosing the appropriate setting.

##### Talking About the Content

Staff talked about the theme of spirituality with students. One trainer reported that they reflect “together on existential questions and needs that come forth when one is seriously mentally ill,” especially if the person was admitted by coercion due to the nature of the illness. The students can also consider the way ahead for the patient moving forward after a period of illness since recovery is the goal for these patients with mental health problems.

##### Choosing the Appropriate Setting

As for training in the clinical setting, the environment was noted as being very important. It is not appropriate to discuss such private things as spiritual concerns in a common room or where others can overhear the conversation; it would be better to talk with the patient in his or her room if the topic is important for them. One person also noted in a final comment that “Religion and life view are and shall not be a theme in the common areas of the ward as this, from experience, might cause discussions, misconceptions, disagreement.” Students are encouraged to learn about and become familiar with the spiritual domain so that they are more comfortable with the topic and can address it with their patients when a patient raises such issues.

## Discussion

### Usability of the Tool in Mental Health

The overall Cronbach *alpha* score (0.838) of the Tool among this sample of mental health staff is high, though not as high as the original testing of the Tool (0.910), which is published elsewhere. Factor analysis of the survey competencies showed that one question in each competency loaded with a very low score due to those questions addressing more than one concept or construct related to spiritual care. Since the goal of this pilot study was to evaluate its use among mental health staff rather than students, we encourage further development and modification of the Tool by its developers. These items reveal a weakness in the Tool that its developers will no doubt need to address at some future date.

The Cronbach *alpha* for this pilot was fairly high, which was consistent with its original parametric testing, demonstrating the overall reliability of this spiritual self-assessment tool. There was a small positive gain when the lowest scoring items were removed, which is a point for the developers to consider as they examine the use of the Tool in a variety of settings. It is interesting to note that scores for the questions on Skills remain strong and are well understood across all competencies. The weakest questions on Attitudes in Competency 1 and 3 actually had multiple concepts within one question, making it hard to choose the best answer. Knowledge scored the lowest among the attitude, skills and knowledge subsets, which is not surprising when you note that very few of the participants had any further training that would help prepare them for spiritual care (see Table 1). Again, question 2 in both Competency 2 and 4 are complex questions, the first asking about awareness and impact, and the second about needs and resources, both of which are multi-faceted. Questions where the primary focus is hard to identify or those that include more than one concept or construct are difficult to answer in any survey. Modification of an instrument, which we recommend for this Tool, is part of an instrument’s development into the most useful tool possible (DeVellis, 2012).

### Implications From Demographic Data

It was important to us that we not include demographic data that could reveal participant identities, so we had only six questions. The issue of gender is not really a useful one since nursing is a female dominated profession, but men tend to move into the higher stress or intensity areas like intensive care, emergency nursing, and mental health (Evans, 1997). With one quarter of our sample being male, this fits into global patterns for nursing staff in mental health. Age and work experience had a strong and significant relationship to each other, which makes sense, but we also found that age and experience approached statistical significance in relation to understanding inter-personal spirituality and assessing patients spiritually. These findings emphasize the importance of personal maturity and work experience with teamwork. Moreover, connecting with others in a collaborative way to accomplish patient-centered spiritual care is critically important in mental health (Herrman, 2017; Milstein et al., 2017; Poncin et al., 2019).

Another interesting element of our sample is that one ward had half the participants. The recruitment efforts were made through the institutional leaders and the leaders of each ward. One ward leader was very interested in the study and encouraged staff on that ward to participate. This emphasizes the role of leadership in research where gatekeepers provide access to participants. Those who become engaged and interested in the project may recruit more participants; moreover, if we want role models in healthcare, leaders also need development (Jenkins et al., 2020). This has implications for researchers, especially in areas where limited studies have been conducted on sensitive topics or with particularly vulnerable people groups.

### Educational Preparation for Spiritual Care

Most of the staff indicated a lack of training related to the spiritual domain. The qualitative findings revealed the need for raised awareness about spiritual care and especially regarding how to develop discernment of how and when to talk with patients in ways that could be supportive, even when patients are delusional or in crisis. The fear of harming mental health patients by opening up for or addressing spiritual and religions matters is well known from the literature (Koslander et al., 2009; Ouwehand et al., 2014; Borge and Maeland, 2017; Neathery et al., 2020). Health care staff in mental health hospitals who are working with very ill patients have an ethical and professional duty to develop knowledge, skills, and attitudes to assess and address their patients also in the area of spirituality; this domain is an aspect of whole person patient-centered care (Saleem et al., 2014; Medås et al., 2017; Patterson et al., 2018). In discerning how to assess patients spiritually, Koslander et al.’s (2020) advises mental health nurses that spirituality can be both a resource and a challenge and is worth noticing, acknowledging, and discussing among the healthcare team members. In a Scandinavian context, it might also be helpful to try to determine if patients have a restful religiosity that is deeply integrated into the person and thus might provide support in times of illness, or if there is some conflict related to their belief system. Crises religiosity, where illness might lead to increased religiosity has not proven to give the same comfort and health benefits as a steady faith or belief system (Hvidt et al., 2017, 2020).

This pilot study revealed that mental health staff with a bachelor’s degree had a higher understanding of spiritual assessment and care even though just three reported to have had any training in spiritual care assessment. To interpret these fining is challenging as more staff might have had some training, but just did not remember it or they might not have considered it spiritual in nature. The high comfort with interpersonal spirituality may indicate that nursing education does a very good job of teaching nursing students therapeutic communication techniques as well as about the value of providing compassionate care and working together to give the best possible patient care. However, the findings do indicate a need for universities to prepare students in a way so that nurses can feel better prepared and that staff can be better role models for students (Kuven and Giske, 2019). Only five out of 17 participants who responded to the request for comments said that they supervised nursing students in assessing patients spiritually during their clinical studies in mental health. With the discernment needed for doing this well, these results call for an increased focus on supervision in spiritual care during students’ placements and for more training that will integrate the spiritual domain into mental health care (Grams et al., 2007).

### Continued Education in Nursing

The results showed that the better the staff know themselves, own beliefs and values, the more able they were to facilitate spiritual care for patients and too evaluate if the care given of help to patients or not. The importance of this finding cannot be overstated. Nurse educators and clinical leaders need to make sure that staff have the opportunity to learn and encouragement to grow within themselves in order to be more able to help others in the spiritual domain.

### Limitations

In addition to this pilot having a small sample size, which limits the ability to find statistically significant differences and/or relationships between survey items, the study was carried out at one site where there was not an evenly distributed number of people from each ward in the sample. Moreover, a factor analysis of the Tool itself, while indicating a strong reliability score that shows its relationship to the constructs of spirituality and spiritual care, demonstrates a need for modification and simplification of some questions. Additionally, qualitative data revealed some redundancy among the questions as well as a lack of clarity on the main point of some questions. Finally, survey tools rely on self-report, which has implicit bias.

## Conclusion

Spirituality and spiritual care are important topics to address in mental health; however, there is limited research that addresses spiritual issues in mental health nursing. This pilot study evaluated the Tool for use among mental health nurses in a mental hospital setting, and the findings support that the Tool can be helpful in determining spiritual care competencies among mental healthcare staff. The caring attitudes and communication skills reflected in the responses is clear evidence that good nursing is whole person and patient centered. This pilot reveals a need for spiritual care educational materials that are targeted specifically for those who work in mental health, materials that address the approach of improving Attitudes, enhancing Skills, and increasing Knowledge related to spirituality and spiritual care of mentally ill patients. Moreover, for a relatively small pilot, the study demonstrated significant findings on what mental health personnel need to know and how they can better support their patients spiritually.

## Data Availability Statement

The raw data supporting the conclusion of this article will be made available by the authors, without undue reservation.

## Ethics Statement

The studies involving human participants were reviewed and approved by Norwegian Protected Data Services. Written informed consent for participation was not required for this study in accordance with the national legislation and the institutional requirements.

## Author Contributions

TG and PC: research design and planning, qualitative data analysis, and manuscript writing. TG: data collection. PC: quantitative data analysis. Both authors agreed to the final manuscript.

## Conflict of Interest

The authors declare that the research was conducted in the absence of any commercial or financial relationships that could be construed as a potential conflict of interest.

## Publisher’s Note

All claims expressed in this article are solely those of the authors and do not necessarily represent those of their affiliated organizations, or those of the publisher, the editors and the reviewers. Any product that may be evaluated in this article, or claim that may be made by its manufacturer, is not guaranteed or endorsed by the publisher.

## Footnotes

1
www.epicc-network.org
